# Effectiveness of Information and Communication Technology–Based Cognitive Behavioral Therapy Using the Smart Sleep App on Insomnia in Older Adults: Randomized Controlled Trial

**DOI:** 10.2196/67751

**Published:** 2025-06-25

**Authors:** ChanHee Kim, Yeonhee Lee, Seung-Gul Kang, Seon-Heui Lee

**Affiliations:** 1 Department of Nursing Graduate School Yonsei University Seoul Republic of Korea; 2 Graduate School Gachon University Incheon Republic of Korea; 3 Department of Psychiatry Gil Medical Center Gachon University College of Medicine Incheon Republic of Korea; 4 College of Nursing Research Institute of AI and Nursing Science Gachon University Incheon Republic of Korea

**Keywords:** insomnia, cognitive behavioral therapy, community health nursing, mobile applications, information communication technology, Smart Sleep, older adults, elderly, sleep disorders, therapy, mhealth, applications, smartphones, sleep quality, randomized controlled trial

## Abstract

**Background:**

Insomnia is a common sleep disorder, especially among older adults, with a significant impact on the quality of life (QoL) and is associated with various comorbidities. Traditional pharmacotherapy for insomnia is often unsuitable for older adults because of potential drug interactions and side effects, making nonpharmacological interventions such as cognitive behavioral therapy for insomnia (CBT-I) more appropriate. However, delivering CBT-I in a traditional face-to-face setting poses challenges including accessibility and adherence, particularly for older adults.

**Objective:**

This study aimed to evaluate the effectiveness of an information and communication technology (ICT)–based CBT-I program, “Smart Sleep,” specifically designed to improve insomnia among community-dwelling older persons.

**Methods:**

A single-blind randomized controlled trial was conducted with 59 older participants from Incheon, South Korea. Participants were divided into an intervention group, which used the Smart Sleep mobile app, and a control group. The intervention group received 8 weeks of non–face-to-face CBT-I through the app, which included sleep diaries, relaxation exercises, and real-time consultations. Outcomes were measured at baseline, week 4, and week 8, with a focus on insomnia severity, sleep quality, sleep efficiency, dysfunctional beliefs about sleep, depression, and QoL.

**Results:**

The intervention group showed significant improvements in insomnia severity, sleep quality, sleep efficiency, and dysfunctional beliefs about sleep compared to the control group. However, there was no significant difference in the QoL between the 2 groups (*F*_2,114_=0.998, *P*=.37). Participation rates in the Smart Sleep program were high, with a 94% completion rate for sleep diary tasks and 100% participation in real-time consultations. In addition, subgroup analysis based on sleep medication use showed significant improvements in insomnia severity for both medicated and nonmedicated participants.

**Conclusions:**

The ICT-based CBT-I program “Smart Sleep” effectively improved sleep-related outcomes among older participants, demonstrating the potential of non–face-to-face interventions in managing insomnia in this population. The program is user-friendly, and ICT-based coaching contributed to high engagement. To ensure broader access for older adults, distribution through community welfare or public health centers is recommended.

**Trial Registration:**

Clinical Research Information Service KCT0007287; https://cris.nih.go.kr/cris/search/detailSearch.do?seq=23344

## Introduction

Insomnia, defined as difficulty with falling asleep or maintaining sleep throughout the night [1], is a prevalent public health issue across a variety of age groups worldwide. This condition is often unrecognized or underreported and is considered a disease with significant economic costs [2]. The prevalence of insomnia increases significantly with age, with studies reporting a chronic insomnia prevalence of 20% to 40% among individuals aged 65 years and older [3]. Insomnia is associated with a high comorbidity with other physical and mental disorders, exhibiting a bidirectional relationship in which each can influence the other [4]. In particular, insomnia in older adults can increase the risk of falls, as well as the onset and worsening of neuropsychiatric disorders, such as depression and dementia, and chronic diseases, such as cardiovascular disease, diabetes, and obesity, thereby reducing the overall quality of life (QoL) [5]. Considering these factors, insomnia in older adults can ultimately have a negative impact on their overall health, necessitating appropriate treatment.

The most common treatments for insomnia include medications and cognitive behavioral therapy for insomnia (CBT-I) [6]. Pharmacological treatments are widely used for insomnia in clinical settings. However, pharmacotherapy may not be appropriate for older people or those with various comorbidities because of drug-drug interactions and side effects [7]. Research has shown that long-term benzodiazepine use in older adults is associated with an increased risk of falls, hip fractures, and cognitive decline [8-10]. Considering that the older population may be more sensitive to drug side effects than other age groups, it is especially important to focus on nonpharmacological approaches to treating insomnia [11]. According to insomnia treatment guidelines, sleep medicine researchers strongly recommend nonpharmacological treatments, particularly CBT-I [12].

CBT-I aims to change dysfunctional thoughts, attitudes, and sleep-related habits. It consists of sleep restriction, stimulus control therapy, relaxation techniques, and sleep hygiene education [13]. Unlike drug therapy, in which patients passively follow a treatment regimen, CBT-I requires patients to actively recognize and assess their problems and engage in the process [14]. For effective treatment, it is essential that patients adhere to the treatment schedule and faithfully follow the recommended sleep-related measures. However, there are practical challenges in providing CBT-I to insomnia patients, such as participant adherence, costs, and time and space constraints [15].

To address these cost and accessibility issues, information and communication technologies (ICTs) can be used to provide therapeutic tools such as CBT-I, enabling the delivery of these interventions in a non–face-to-face manner. Using ICT, it is possible to overcome the limitations of time and space, making it easier for older adults, who may have difficulty visiting clinics multiple times, to receive treatment [15]. In addition, ICT facilitates real-time monitoring and feedback to participants, which can help enhance patient engagement [16]. Previous studies have demonstrated the effectiveness of internet- or telephone-based CBT-Is in adults with insomnia [17-20]. However, to the best of our knowledge, few programs have specifically applied mobile app–based non–face-to-face CBT-I to improve insomnia among community-dwelling older people. Therefore, this study aimed to implement and evaluate the effectiveness of an ICT-based CBT-I program, “Smart Sleep” developed for older individuals with insomnia in the community.

## Methods

### Study Design and Setting

This randomized, single-blind, controlled study was conducted between May 2022 and October 2022 in Incheon Metropolitan City, Republic of Korea (Multimedia Appendix 1).

### Ethics Approval

This study was approved by the Gachon University institutional review board (1044396-202203-HR-068-01) and registered in the Clinical Research Information Service (KCT0007287). Approval from the institutional research ethics committee was obtained before the experiment began, and the study was conducted in accordance with the principles set forth in the Declaration of Helsinki. Participants were briefed on the purpose of the study and the measures taken to protect their privacy before the study commenced. All participants provided written informed consent. Participants received a stipend of KRW 10,000 (US $7.34) for each study visit (baseline, 4 weeks, and 8 weeks) as compensation for their time and participation.

### Participants

A total of 100 participants were enrolled in this study between May 2022 and July 2022. We included community-dwelling older people older than 60 years of age living in Incheon City, and those scoring 8 or higher on the Insomnia Severity Index (ISI) questionnaire [21] were selected as subjects. Participants were recruited through a local public health center, where individuals were registered for the home-visit nursing care program. The exclusion criteria included people with serious neuropsychiatric problems, hearing or seeing difficulties, a history of alcohol or drug abuse, or diseases and disabilities that could hinder participation in the exercise program.

Of the 100 patients, 25 did not consent to participate in the study, 1 was excluded due to difficulty in walking, 10 were excluded due to a score of less than 8 on the insomnia scale, and 4 were excluded for personal reasons. Among the 25 individuals who declined participation, the most commonly cited reasons were lack of time due to personal schedules, limited interest in participating in research, and concerns about potential increases in electricity costs associated with using the app. Finally, 60 patients were included in the study. On the first day of the study, 1 participant in the control group was excluded for personal reasons and expressed refusal. Ultimately, the intervention was conducted on 30 participants in the experimental group and 29 participants in the control group.

G*Power (version 3.1.9.7; Helsinki Heine University) software was used to determine the sample size. Following the Cohen method, a medium effect size (0.25) was selected, which is appropriate for behavioral intervention studies. The alpha error probability and power were set at .05 and .95, respectively, resulting in a required sample size of 44 participants. In addition, considering that the study participants are older adults and the potential impacts of the COVID-19 pandemic, a natural attrition rate of 25% was accounted for. This calculation reflects the study’s focus on older adults and the adaptations made to the CBT-I protocol, including moderated sleep restrictions tailored to their needs.

### Randomization and Blinding

Simple randomization and hidden assignments were performed using a computer-generated randomization table before data collection. Participants were randomly classified into two groups: intervention and control groups. The order of the assignments was hidden from the participants until the intervention was complete. To reduce bias, controls were also provided with tablets (Lenovo, 10.1, 1920×1200) and smart bands (Story4you, Korea) and were trained on how to use basic devices.

### Intervention

The intervention provided CBT to older people with insomnia using a Smart Sleep app for 8 weeks. The app was developed following a systematic process, including content determination through a systematic review and preference survey, interface design, and iterative usability testing. Key features, such as sleep information, habit improvement, and real-time counseling, were finalized based on preference analysis and expert feedback. These steps are detailed in a separate published manuscript [22,23].

Preliminary training was conducted for 5 days before the intervention. All conditions were identical between the groups, except for the program, which ensured that the control group received the same conditions as the intervention group. This included training in tablet use, internet access, and provision of digital devices (tablets and smart bands) with limited content on today’s to-do tasks to the control group. During the intervention, non–face-to-face services were used for the experimental group for 8 weeks to manage the participants. Questions and answers from users were answered, and participation was encouraged.

The control group received usual care at a public health center. After all interventions and outcome measures were completed in the experimental group, the wait-listed participants in the control group received the intervention. To minimize potential contamination between the intervention and control groups, participants were assigned to different time slots and locations for their respective sessions. They were also instructed not to discuss the study details or their participation with individuals outside their group. These steps were taken to reduce the likelihood of information-sharing or cross-interaction.

The main program content consisted of sleep information (sleep diary), relaxation training, sleep-related videos, exercise, and real-time consultation (Table 1). All contents were set and managed in today’s to-do list according to their frequency.

Sleep information was collected and an appropriate sleep plan was presented to each individual. Based on the sleep information collected through the sleep diary and sleep band, such as the time the participant went to bed, the time they actually fell asleep, the time they woke up in the morning, and the time they fully woke up, the sleep time appropriate for the participant was presented on the My Sleep Plan screen of sleep management. The participants completed a sleep diary every day for 8 weeks to obtain subjective sleep data. Sleep activity records were automatically uploaded and managed daily on the administrator’s screen by having patients wear a sleep band.

The videos included relaxation training and educational videos. The participants were encouraged to watch relaxation and abdominal breathing training videos daily. In addition, in the sleep helpers category, meditation, sounds of nature, and sleeping music were included to help participants sleep with peace of mind. The educational videos included videos on sleep and insomnia, healthy sleeping habits, insomnia treatment, and relaxation training. By watching educational videos and providing accurate information about sleep through real-time education, sleep awareness was improved. For real-time exercise, participants were asked to engage twice a week in silver gymnastics and a walking program conducted at the public health center. In addition, one of the regular exercises was to walk more than 7000 steps per day. The number of steps was measured using a sleep band and uploaded to the manager’s program. To ensure safety, the mobile app provided video demonstrations. Participants were advised to consult their health care providers before starting the program. Further safety measures included recommending seated exercises for those experiencing discomfort and encouraging caregiver assistance when needed.

The researchers monitored participants’ engagement and encouraged active participation, with the program content provided exclusively to the intervention group. When today’s to-do tasks were not implemented, the manager delivered a push notification to the target person so that they could perform today’s to-do tasks. In addition, feedback was provided to participants at least once a week using video or phone calls connected to the mobile app. Live classes included consultations and training. Through real-time consultation and education, we communicated with researchers and developed knowledge about sleep to correct cognitive distortions. In addition, pre-registered participants, along with their families and friends, were allowed video calls to provide emotional support.

**Table 1:** Contents of the CBT-I program “Smart Sleep.”

Contents of program	Program frequency	Description
Sleep diary	Daily	Self-reporting information about last night's sleep
Sleep band	Daily	Collecting sleep information wearing a sleep band
Survey	Once a week	Self-reporting the insomnia questionnaire using the ISI-K^a^ Self-reporting the sleep habits questionnaire
Sleep management	Daily	Based on sleep information by diary, band, and survey, providing recommendations on appropriate sleep duration for sleep restriction
Relaxation training video	Daily	Providing video for relaxation technique
Sleep helper	Daily	Equipped with sleep-aiding meditation, sleep music, and nature sounds
Sleep-related educational video	Daily	Providing video about correct information about sleep, proper sleep hygiene, sleep stimulus control methods, etc
Exercise	Daily	Offering a real-time exercise program twice a week and a fall prevention exercise video and providing a pop-up notification to encourage walking 7000 steps
Today’s to-do list	Daily	Sending push notification for incomplete tasks. Applying goal achievement stamp for completed tasks
Real-time training and consulting	Once a week, with additional sessions upon request	Based on sleep information by diary, band, and survey, providing personalized sleep consultation and improving sleep-related cognition

^a^ISI-K: Korean version of the Insomnia Severity Index.

### Outcome Measures

All assessments were performed in the same week for both the intervention and control groups at baseline (week 0) and weeks 4 and week 8. We collected data through a self-administered survey conducted when the participants visited the public health center.

The primary outcome of this study was insomnia severity, assessed using the Korean version of the Insomnia Severity Index (ISI-K). Secondary outcomes included quality of sleep, sleep efficiency, dysfunctional beliefs and attitudes about sleep, depression levels, and quality of life.

#### Insomnia Severity

The ISI, an insomnia severity scale, was adapted by Cho [24] from the tool developed by Bastein [21]. The ISI consists of 7 items rated on a 5-point Likert scale, with total scores ranging from 0 to 28. Higher scores indicate more severe insomnia.

#### Quality of Sleep

Sleep quality was measured using the Pittsburgh Sleep Quality Index (PSQI) [25], a self-report measure of perception of habitual sleep translated by Sohn [26] into the Korean version of the PSQI (PSQI-K). The PSQI-K comprises 19 items. The total score ranges from a minimum of 0 to a maximum of 21, with higher scores indicating poorer sleep quality.

#### Sleep Efficiency

Sleep efficiency is calculated as a percentage by dividing the total sleep time by the total time spent in bed and is the most common indicator for objectively evaluating sleep quality. Total sleep time was calculated as the total time spent in bed minus sleep latency and awakening time after sleep [27,28]. In our program, sleep efficiency was calculated based on sleep diaries.

#### Dysfunctional Beliefs and Attitudes About Sleep

Dysfunctional Beliefs and Attitudes About Sleep (DBAS) is a self-report scale with a total of 16 questions developed to evaluate insomnia patients’ incorrect beliefs and attitudes about sleep developed by Morin [29]. This study used the Korean version of the DBAS (K-DBAS-16), adapted and validated by Yoo et al [30]. The lower the score on the DBAS, the more positive results can be inferred from CBT-I.

#### Depression

The depression scale translated by Cho et al [31] was used, based on the Geriatric Depression Scale (GDS) [32]. The total score is 15 points; the higher the score, the higher the level of depression.

#### QoL

EQ-5D is a tool developed by the EuroQol Group to measure health-related QoL and consists of 5 items: exercise ability, self-care, daily activities, pain, inconvenience, anxiety, and depression [33].

### Statistical Analysis

The collected data were analyzed using SPSS WIN 28.0 (IBM Corp). The general characteristics of participants were analyzed using the chi-square and *t* tests. Intention-to-treat analysis was applied, which includes all participants as originally assigned, regardless of whether they completed the intervention. The normality of each variable was tested using the Shapiro-Wilk test, a statistical method to determine whether a dataset follows a normal distribution. All indicators were found to be normally distributed and were analyzed using an independent *t* test.

The results of the app usability measurement conducted in the eighth week for the experimental group were analyzed using means and SD. To analyze the mean differences between groups and over time, repeated measures ANOVA was conducted on sleep quality, depression, QoL, dysfunctional beliefs and attitudes toward sleep, insomnia, and sleep efficiency. A paired *t* test was used to compare changes in sleep quality, depression, QoL, dysfunctional beliefs and attitudes toward sleep, insomnia scale, sleep efficiency, and baseline scores between the experimental and control groups over time. The significance level was set at *P*<.05.

Effect sizes were reported as partial eta-squared (η^2^*p*) to quantify the proportion of variance explained by each effect. Partial eta-squared values were interpreted using the following thresholds: small (0.01), medium (0.06), and large (0.14) as recommended by Cohen [34]. In addition, Cohen *d* was calculated as a standardized effect size to facilitate comparisons with previous studies. It was computed using the mean difference between groups at week 8, divided by the pooled SD. Effect sizes were interpreted as small (0.2), medium (0.5), and large (0.8) based on conventional thresholds [34]. The number needed to treat (NNT) was calculated for the primary outcome, ISI, to assess the clinical significance of the intervention [35]. NNT indicates the number of participants required to treat to achieve one additional remission compared with the control group. Remission was defined as an ISI score<8, consistent with previous studies on CBT-I [36].

## Results

### Participant Characteristics

**Figure 1:**
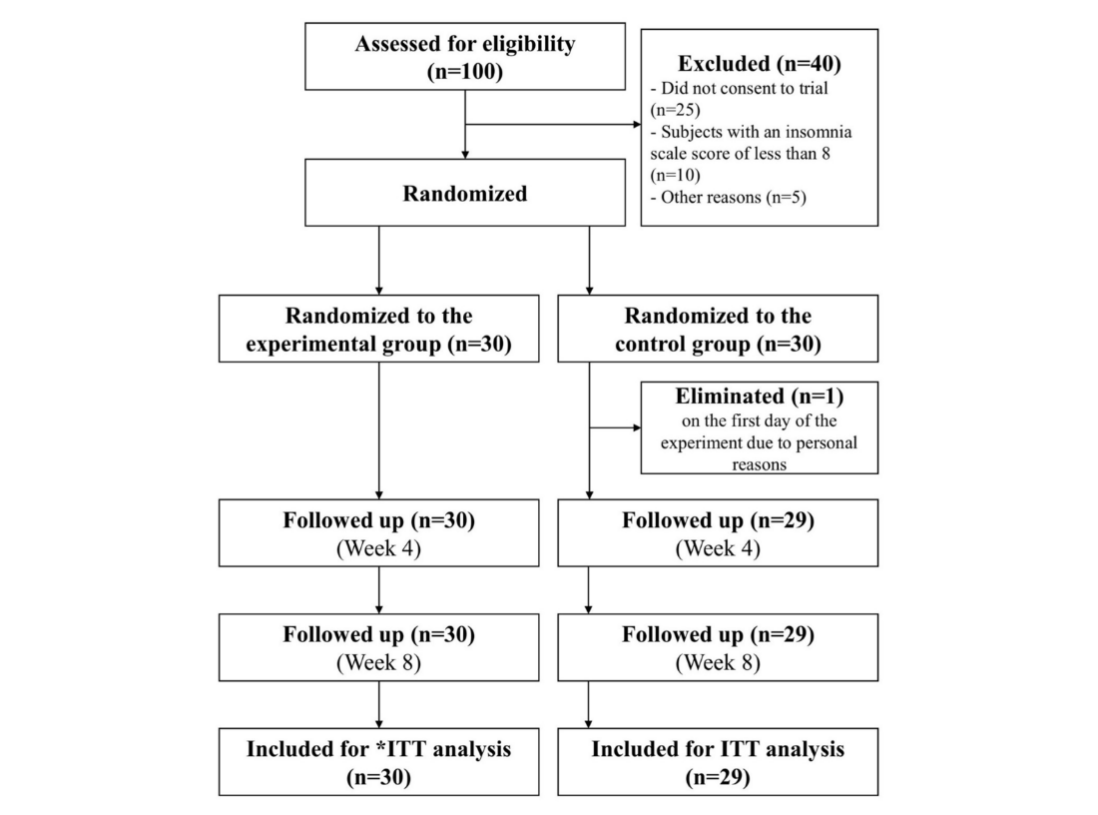
Study flowchart. A total of 60 participants were randomized (30 per group), with one 1 participant in the control group excluded due to personal reasons. Both groups were followed up at weeks 4 and 8, and the final analyses were conducted using the intention-to-treat (ITT) approach, including 30 participants in the experimental group and 29 in the control group. Intention to treat refers to the analysis based on the initial treatment group division, rather than dividing the patient group according to whether the patient actually received treatment.

One of the 60 participants was excluded from the trial for personal reasons after randomization on the first day of the experiment (one from the control group). A total of 59 participants (30 in the experimental group and 29 in the control group) completed the study (Figure 1). All baseline general characteristics were well-matched between the experimental and control groups (Table 2). The average age of the participants was 70.7 (SD 4.49) years in the experimental group and 70.27 (SD 4.97) years in the control group. The experimental and control groups included 6 (20%) men and 24 (80%) women, and 6 (20.6%) men and 23 (79.3%) women, respectively.

**Table 2:** Baseline characteristics of participants.

Parameter					Experimental group (n=30)		Control group (n=29)		*P* value	
Age (years), mean (SD)					70.70 (4.49)		70.27 (4.97)		.59	
**Gender, n (%)**										.75
			Men		6 (20)		6 (20.6)			
			Women		24 (80)		23 (79.3)			
**Education, n (%)**										.29
			Uneducated		1 (3.3)		2 (6.9)			
			Elementary school		8 (26.7)		9 (31)			
			Middle school		10 (33.3)		6 (20.6)			
			High school		8 (26.7)		9 (31)			
			≥College		3 (20)		2 (6.8)			
**Living state, n (%)**										.44
	Alone			25 (83.3)		23 (79.3)				
	With family			5 (16.6)		6 (20.6)				
**Number of drugs, n (%)**										.45
		≤3			25 (83.3)		26 (89.6)			
		≥4			5 (16.7)		5 (16.7)			
**Smoking, n (%)**										.16
			Yes		2 (6.6)		2 (6.8)			
			No		28 (93.3)		27 (93.1)			
**Drinking, n (%)**										.74
			Yes		3 (10)		4 (13.7)			
			No		27 (90)		25 (86.2)			
**Use of sleeping pills, n (%)**										.23
		Yes			13 (43.3)		10 (34.4)			
		No			17 (56.6)		19 (65.5)			
ISI-K^a^, mean (SD)					14.47 (4.52)		14.93 (6.09)		.51	
Sleep efficiency, mean (SD)					61.25 (16.64)		59.13 (18.75)		.56	
PSQI-K^b^, mean (SD)					11.73 (3.33)		10.24 (3.94)		.92	
K-DBAS-16^c^, mean (SD)					104.4 (32.55)		109.9 (35.45)		.65	
SGDS-K^d^, mean (SD)					6.23 (3.99)		6.65 (4.58)		.27	
EQ-5D, mean (SD)					0.75 (0.16)		0.65 (0.22)		.91	

^a^ISI-K: Korean version of the Insomnia Severity Index.

^b^PSQI-K: Korean version of the Pittsburgh Sleep Quality Index.

^c^K-DBAS-16: Korean version of the Dysfunctional Beliefs and Attitudes About Sleep.

^d^SGDS-K: Korean version of the short geriatric depression scale.

As a result of testing the homogeneity of the research variables before the experiment using a *t* test, there was no significant difference between the experimental and control groups in terms of the main research variables (Table 2). For the insomnia scale, the scores were 14.47 (SD 4.52) points in the experimental group and 17.28 (SD 5.87) points in the control group. Sleep quality was rated at 11.73 (SD 3.33) points in the experimental group and 10.24 (SD 3.94) points in the control group. The recorded sleep efficiency was 61.25 (SD 16.64) in the experimental group and 59.13 (SD 9.88) in the control group.

### Comparison of the Effects Between the Intervention and Control Groups

**Figure 2:**
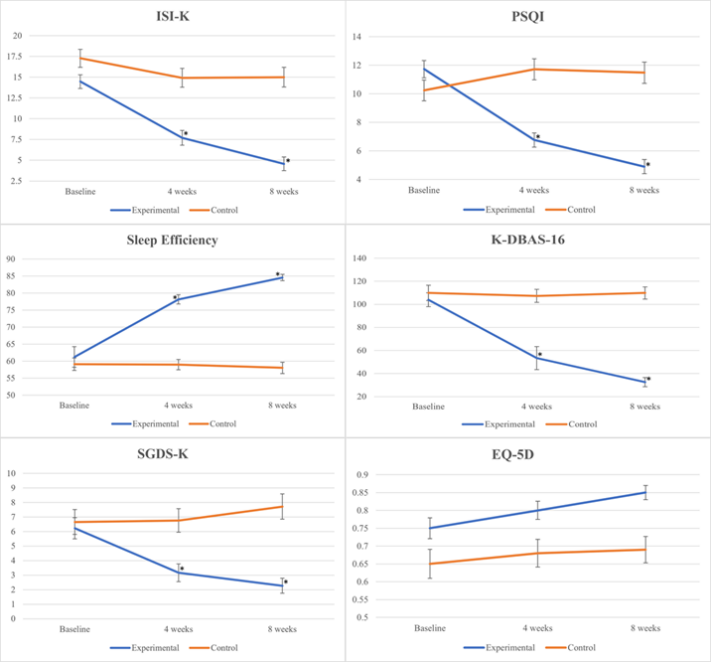
Effect of the intervention on outcome measures at baseline, 4 weeks, and 8 weeks. ISI-K: insomnia severity index; K-DABS: Dysfunctional Beliefs and Attitudes about Sleep; PSQI: Pittsburgh sleep quality index; SGDS-K: short geriatric depression scale. *t test for change from baseline between groups: *P*<.05. Error bars represent the SEM.

Statistically significant differences were observed in the ISI-K, PSQI-K, sleep efficiency, K-DBAS-16, and SGDS-K (the Korean version of the short geriatric depression scale) scores (*P*<.001 for all), whereas the EQ-5D scores (*P*=.37) did not show statistical significance. The control group showed no significant changes in any of the indicators before or after the intervention (Figure 2 and Table 3).

Data analysis revealed the following results regarding the interaction effects between time points and groups. The primary outcome was insomnia severity, which showed a statistically significant interaction (*F*_2,114_=18.923, η^2^*p*=0.25) with a large effect size. The Cohen *d* value was 1.88. Using an ISI score <8 to define remission, the intervention group demonstrated a significantly higher remission rate compared with the control group, reflected in an NNT of 1.37 (95% CI 1.11-1.80). Secondary outcomes also showed significant interactions. Sleep quality (*F*_2,114_=97.806, η^2^*p*=0.632, *d*=1.92), sleep efficiency (*F*_2,114_=22.394, η^2^*p*=0.48, *d*=3.65), DBAS (*F*_2,114_=77.233, η^2^*p*=0.58, *d*=3.05), and depressive symptoms (*F*_2,114_=25.715, η^2^*p*=0.31, *d*=1.41) showed statistically significant interactions between time points and groups (*P*<.001 for all). The partial eta-squared values ranged from 0.249 to 0.632, which are considered large effect sizes [34]. In contrast, QoL scores did not show a statistically significant interaction between time points and groups (*F*_2,114_=0.998, η^2^*p*=0.02, *d*=0.99; *P*=.37). However, there were significant differences between the groups (*F*_1,57_=9.779; *P*=.003) and across time points (*F*_2,114_=5.423; *P*=.006).

**Table 3:** Comparison of the effects between the intervention and control groups.

Outcome measures			Mean (SD)						Change from baseline, mean (95% CI)					Time and group interaction, *P* value	
			Baseline		Week 4		Week 8		Week 4		Week 8				
**ISI-K^a^**															<.001
	Intervention	14.47 (4.52)		7.70 (4.85)		4.57 (4.55)		–6.76^b^ (–9.19 to –4.96)		–9.90^a^ (–12.24 to –7.55)					
	Control	17.28 (5.87)		14.93 (6.09)		15.00 (6.38)		0.00 (–3.20 to 3.20)		–2.27 (–5.50 to 0.94)					
**PSQI-K^c^**															<.001
	Intervention	11.73 (3.33)		6.77 (2.74)		4.90 (2.72)		–4.96^a^ (–6.54 to –3.39)		–6.83^a^ (–8.40 to –5.26)					
	Control	10.24 (3.94)		11.72 (3.98)		11.48 (4.02)		1.48 (–0.60 to 3.56)		1.24 (–0.85 to 3.33)					
**Sleep efficiency**															<.001
	Intervention	61.25 (16.64)		78.16 (7.44)		84.57 (5.06)		16.91^a^ (14.13 to 19.69)		23.32^a^ (21.43 to 25.21)					
	Control	59.13 (9.88)		59.0 (8.06)		58.04 (8.94)		–0.04 (–3.05 to 2.97)		–1.09 (–4.43 to 2.25)					
**K-DBAS-16^d^**															<.001
	Intervention	104.0 (32.56)		53.40 (54.85)		32.58 (21.78)		–51.0^a^ (–65.96 to –36.03)		–71.86^a^ (–86.18 to –57.55)					
	Control	109.93 (35.45)		107.28 (30.43)		109.83 (28.5)		–2.65 (–20.03 to 14.72)		–0.10 (–17.02 to 16.81)					
**SGDS-K^e^**															<.001
	Intervention	6.23 (3.99)		3.17 (3.36)		2.27 (2.83)		–3.06^a^ (–4.97 to –1.15)		3.96^a^ (–5.75 to –2.17))					
	Control	6.66 (4.59)		6.76 (4.37)		7.72 (4.68)		0.1 (–2.25 to 2.46)		1.06 (–1.36 to 3.50)					
**EQ-5D**															.37
	Intervention	0.75 (0.16)		0.80 (0.14)		0.85 (0.11)		0.05 (–0.02 to 0.13)		0.1 (0.03 to 0.18)					
	Control	0.65 (0.22)		0.68 (0.21)		0.69 (0.2)		0.37 (–0.78 to 0.15)		0.04 (–0.06 to 0.15)					

^a^ISI-K: Korean version of the Insomnia Severity Index.

^b^*t* test for change from baseline between groups: *P*<.05.

^c^PSQI-K: Korean version of the Pittsburgh Sleep Quality Index.

^d^K-DBAS-16: Korean version of the Dysfunctional Beliefs and Attitudes About Sleep.

^e^SGDS-K: Korean version of the short geriatric depression scale.

Further analysis of within-group changes showed significant improvements in the intervention group from baseline to week 4 and week 8. In the intervention group, the ISI-K scores showed a statistically significant reduction at weeks 4 (–6.76, 95% CI –9.19 to –4.34) and 8 (–9.90, 95% CI –12.24 to –7.55). Similarly, the PSQI-K scores demonstrated a notable decline at week 4 (–4.96, 95% CI –6.54 to –3.39) and further at week 8 (–6.83, 95% CI –8.40 to –5.26) from the baseline. In terms of sleep efficiency, there was a significant increase at weeks 4 (16.91, 95% CI 10.25-23.58) and 8 (23.32, 95% CI 16.86-29.68). The K-DBAS-16 scores also showed a considerable drop, with week 4 recording a decrease of –51.0 (95% CI –65.96 to –36.03) and week 8 showing a further reduction to –71.86 (95% CI –86.18 to –57.55). Finally, the SGDS-K scores were significantly lower by week 4 (–3.06, 95% CI –4.97 to –1.15) and continued to decrease by week 8 (–3.96, 95% CI –5.75 to –2.17) compared with the baseline. Although the EQ-5D scores tended to increase at week 8 (0.10, 95% CI 0.03-0.18) from the baseline, the difference between the 2 groups was not statistically significant. In the within-group comparison before and after the intervention, the experimental group showed a significant difference at 8 weeks (*P*=.006), whereas the control group did not (*P*=.44).

### Subgroup Analysis: Participation Rate and Sleep Medication Use in the Experimental Group

Participation rates in the Smart Sleep program were high, with a 94% completion rate for sleep diary tasks and 100% participation in real-time consultations.

Differences in outcome measures according to the participation rate in the Smart Sleep app program were investigated. In the experimental group, participants were divided into high- and low-participation groups based on a 70% total participation rate in the Smart Sleep program. Neither group followed a normal distribution; therefore, the Wilcoxon test was conducted. The results showed that changes in the ISI-K, PSQI-K, sleep efficiency, K-DBAS-16, and SGDS-K scores before and after the intervention were significant in the group with high participation rates. Participants with a participation rate of 70% or higher showed an improvement in their QoL, while the EQ-5D results did not significantly change in the group with lower participation rates after the intervention (Table 4).

**Table 4:** Subgroup analysis according to program participation in the intervention group.

Outcome measures and group		Baseline, mean (SD)	Week 8, mean (SD)	Comparison by time in group, z (*P* value)
**ISI-K^a^**				
	High (n=20)	14.2 (4.50)	4.25 (4.15)	–3.826 (<.001)
	Low (n=10)	15.00 (4.76)	5.20 (5.43)	–2.805 (.005)
**PSQI-K^b^**				
	High	11.60 (3.66)	4.95 (2.62)	–3.931 (<.001)
	Low	12.00 (2.70)	4.80 (3.04)	–2.814 (.005)
**Sleep efficiency**				
	High	61.25 (18.33)	85.00 (4.54)	–3.824 (<.001)
	Low	60.90 (13.16)	83.90 (6.22)	–2.805 (.005)
**K-DBAS-16^c^**				
	High	100.80 (32.99)	31.85 (24.19)	–3.920 (<.001)
	Low	111.60 (32.11)	33.90 (17.01)	–2.803 (.005)
**SGDS-K^d^**				
	High	4.90 (3.38)	1.40 (1.84)	–3.768 (<.001)
	Low	8.90 (3.90)	4.20 (3.61)	–2.809 (.005)
**EQ-5D**				
	High	0.78 (0.14)	0.87 (0.12)	–2.741 (.003)
	Low	0.68 (0.19)	0.81 (0.07)	–1.886 (.059)

^a^ISI-K: Korean version of the Insomnia Severity Index.

^b^PSQI-K: Korean version of the Pittsburgh Sleep Quality Index.

^c^K-DBAS-16: Korean version of the Dysfunctional Beliefs and Attitudes About Sleep.

^d^SGDS-K: Korean version of the short geriatric depression scale.

Participants in the experimental group were divided into high- (≥70%) and low-participation (<70%) groups. Changes in ISI-K, PSQI-K, sleep efficiency, K-DBAS-16, SGDS-K, and EQ-5D scores are presented, with significant improvements observed in the high-participation group.

Among the 30 participants in the experimental group, 17 were taking sleep medications, while 13 were not (Table 5). A subgroup analysis based on sleep medication use was conducted to examine pre-post changes within each group. The results indicated that both the medication and nonmedication groups demonstrated significant improvements across all measured outcomes from baseline to week 8. For insomnia severity, paired *t* tests revealed statistically significant reductions in scores for the medication group (*t*_16_=10.616; *P*<.001) and the nonmedication group (*t*_12_=6.159, *P*<.001). The effect size (*d*) for ISI-K was 0.59. Similar patterns were observed for other variables, including PSQI-K (*d*=0.87) and K-DBAS-16 (*d*=0.90).

Participants were divided into “Yes” (taking sleep medication) and “No” (not taking sleep medication) groups. Both groups demonstrated significant improvements in ISI-K, PSQI-K, and K-DBAS-16 scores from baseline to week 8, regardless of medication use.

**Table 5:** Subgroup analysis according to sleep medication use in the intervention group.

Outcome measures and group		Baseline, mean (SD)	Week 8, mean (SD)	Comparison by time in group, *t* (*df*; *P* value)
**ISI-K^a^**				
	Yes (n=17)	12.35 (2.34)	3.41 (3.53)	10.616 (16; <.001)
	No (n=13)	17.23 (5.25)	6.08 (5.39)	6.159 (12; <.001)
**PSQI-K^b^**				
	Yes	10.12 (2.57)	3.94 (2.41)	7.757 (16; <.001)
	No	13.85 (3.08)	6.15 (2.67)	10.435 (12; <.001)
**K-DBAS-16^c^**				
	Yes	88.18 (29.03)	24.47 (15.98)	8.934 (16; <.001)
	No	125.62 (24.04)	43.08 (24.36)	9.671 (12; <.001)

^a^ISI-K: Korean version of the Insomnia Severity Index.

^b^PSQI-K: Korean version of the Pittsburgh Sleep Quality Index.

^c^K-DBAS-16: Korean version of the Dysfunctional Beliefs and Attitudes About Sleep.

## Discussion

### Effects of ICT-Based CBT-I on Insomnia

This study confirmed that a mobile app–based CBT-I program is highly effective in improving sleep in older people. Significant improvements were observed in sleep-related indicators, including insomnia severity, sleep efficiency, sleep quality, and DBAS, in the intervention group compared with the control group.

The significant improvement in the ISI scores was consistent with previous CBT-I studies on the older using a telephone- or internet-based program [17,18,34]. However, to the best of our knowledge, only a few studies have examined CBT-I programs using ICTs (mobile or digital CBT-I) that are more advanced than web-based CBT-I in older adults. A case report of mobile app–assisted CBT-I for older adults in Taiwan reported that a 64-year-old woman successfully quit sleeping pills, and her sleep quality, evaluated using a Likert scale, improved after using a mobile app [37]. In addition, a pre-post comparison study using a mobile app in Korea found that changes in subjective sleep quality based on the PSQI after the intervention were significant [38]. Although this study was not an RCT, it demonstrated the possibility that a mobile phone–based self-help CBT-I app could be useful in improving sleep in older adults [38]. Our study demonstrated an effect size (η^2^*P*=.249, *d*=1.88, categorized as large) for reducing insomnia severity, which aligns with the large effect sizes (*d*=0.21~1.09) reported in a review study [39] for digital CBT-I interventions. These findings indicate that ICT-based CBT-I can deliver clinically meaningful outcomes similar to those of traditional face-to-face CBT-I (*d*=0.98, classified as large). Furthermore, the NNT was calculated as 1.37 (95% CI 1.11-1.80), meaning that approximately 1-2 participants would need to be treated with this program to achieve one additional remission of insomnia. Given that our study specifically focused on older adults, these findings suggest that digital interventions could help address the unique needs of this population, who often face challenges in accessing traditional face-to-face therapy.

In this study, sleep restriction was somewhat more flexible than in the general population, considering the characteristics of older users, which may have helped to keep participants more engaged. The use of a moderated sleep restriction approach specifically tailored for older adults led to improvements in sleep-related measures, even among the older people targeted in this study. Older adults may struggle with strict components of CBT-I, such as sleep restriction, due to the physical and cognitive demands of aging [40]. Considering the characteristics of the older with reduced adaptability to abrupt changes in sleep patterns [41], sleep restriction was not excessively enforced, but was gradually adjusted to improve sleep efficiency at an appropriate pace. While previous studies have set the sleep efficiency target for adults at 85% [42,43], we adjusted the target to 75% to account for the unique sleep characteristics of older individuals.

In addition, by combining the subjective sleep diary, which participants filled out themselves, with objective sleep data collected from wearable devices, more appropriate sleep restrictions could be applied to each individual. Using a combination of subjective and objective sleep data may help correct sleep misperceptions in older adults, allowing for a more accurate sleep plan. Both the ISI and PSQI are widely validated tools for assessing insomnia severity, treatment response, and sleep quality, respectively, but they rely on participants’ subjective perceptions of sleep [44]. This reliance may introduce potential biases, particularly in older adults, due to cognitive decline or varying interpretations of survey items. However, the integration of wearable device data in this study effectively mitigated such biases, providing a more reliable and comprehensive evaluation of sleep patterns. Older adults tend to overestimate or underestimate their actual sleep duration, making it difficult to accurately assess sleep problems based solely on subjective data [45]. Therefore, this study supplemented subjective perceptions by rechecking objective data from a wearable device, leading to more accurate sleep prescriptions and effective outcomes for insomnia. Significant improvements were observed in the ISI scores and sleep efficiency among participants in our study, which is consistent with the findings of other studies conducted in adult populations [46,47].

Other possible reasons why the treatment program used in this study might have been effective include various elements based on CBT-I, such as sleep diaries for sleep stimulus control and sleep hygiene improvement, non–face-to-face counseling, sleep management for sleep restriction, and exercise programs for relaxation training, which are expected to have contributed to the systematic improvement of participants’ sleep patterns and perceptions about sleep. Recent evidence [48] emphasizes that sleep stimulus control and sleep restriction are highly effective components of CBT-I, which aligns with the approach used in this study. While they caution that certain relaxation techniques may be counterproductive [48], the structured exercise program in this study could have supported relaxation in a controlled and beneficial way, complementing other CBT-I elements. This suggests that the integration of well-designed exercise routines into CBT-I programs could enhance their overall effectiveness when tailored appropriately.

Moreover, cognitive restructuring through cognitive therapy components, sleep stimulus control methods, and sleep hygiene in our treatment program may have led to improvements in the dysfunctional beliefs scored using the DBAS scale. A previous mobile CBT-I study did not show a significant improvement in the DBAS scores, possibly because of its sole focus on sleep restriction [46]. In contrast, our multifaceted CBT-I approach incorporated cognitive therapy alongside sleep stimulus control and sleep hygiene practices, which may have contributed to these positive outcomes. In our study, sleep stimulus control was adjusted to suit the adaptability of the older adults. While recommendations for adults suggest leaving the bed after 15-20 minutes of being awake [43], we modified the protocol to allow up to 30 min before instructing the participants to leave the bed. This adjustment aimed to provide a more flexible approach that was better suited to the needs of older individuals, ensuring that they could adapt to changes more comfortably while maintaining the principles of stimulus control therapy. In addition, daily reinforcement of sleep hygiene practices and non–face-to-face counseling provided participants with ongoing support, likely enhancing the intervention’s overall effectiveness. These findings emphasize the value of incorporating cognitive therapy into CBT-I programs to address dysfunctional beliefs, particularly in older populations.

### Effects of ICT-Based CBT-I on QoL

The results of this study showed that QoL improved in the intervention group before and after the intervention, whereas the control group did not show a statistically significant difference. However, when comparing the intervention and control groups, there was no significant difference in the improved QoL. The effect of CBT-I on QoL has shown varying results across studies, with some finding it effective and others not [22,49]. QoL is a multidimensional concept comprising physical, mental, and economic factors. These are assessed through multiple domains in the EQ-5D, such as exercise ability, self-care, daily activities, pain, discomfort, and anxiety and depression [50]. Since CBT-I mainly focuses on improving sleep quality, its impact on other aspects of QoL may be relatively small or indirect. External factors, such as medical comorbidities, physical activity levels, employment status, and emotional well-being, could have diluted the observed effects of insomnia improvement on QoL scores in this study. In particular, older adults have multiple medical and psychiatric comorbidities and environmental factors that are difficult to change; therefore, global QoL improvement from improving insomnia may be limited. Nevertheless, the significant effects observed before and after the intervention in the intervention group, unlike in the control group, suggest that ICT-based CBT-I has the potential to improve QoL. In our study, QoL also improved in the high adherence group (≥70% adherence), suggesting that improving adherence will lead to improved QoL. Future studies should consider how ICT-based CBT-I programs can improve other factors related to the QoL, such as emotional stability, stress management, and social interaction, in addition to sleep.

### Boosting Engagement in Non–Face-to-Face CBT-I: Insights Into Adherence

This study showed a dropout rate of 1.6%, which is lower than the 6% to 48% reported in other studies [51]. In addition, the sleep diary mission completion rate was 94%, and the real-time consulting participation rate was 100%, both of which were very high. Due to cognitive decline, older adults find it easier to record sleep patterns using simple, predefined options rather than writing complex responses [40]. Reflecting on this characteristic of the older, this study designed a sleep diary that proceeds automatically by presenting questions with a simple touch, allowing participants to complete the survey more easily. This could be a key factor in increasing adherence rates among older adults. Although older adults may be slower to adapt to ICT, such as mobile apps, than younger generations, the design of mobile apps with high user-friendliness and appropriate feedback and support, such as telephone coaching, can lead to higher treatment retention rates and better treatment outcomes.

Increasing the participation rate in non–face-to-face interventions is a challenging task [52]. In this study, the analysis of the Smart Sleep app participation rate showed that the group with a high participation rate showed improvement in both the sleep scale and QoL, whereas the group with a low participation rate did not show a significant improvement in the QoL. Therefore, enhancing user involvement is crucial for the effective treatment of insomnia in non–face-to-face environments. This is consistent with an important element of CBT-I, which improves misconceptions about insomnia through the active participation of subjects. In addition, strategies such as goal achievement stamps and incentives to encourage continuous participation are believed to enhance this effect. As this study was based on data limited to a specific population, further research is required on the relationship between program components, participation rates, and outcomes.

### CBT-I and Sleep Medication Use: Toward Sustainable Insomnia Management

In this study, both the sleep medication and nonmedication groups demonstrated significant improvements in insomnia severity and related outcomes over the 8-week intervention period. The effect size for ISI, based on group comparisons, was medium (*d*=0.59), highlighting the meaningful impact of the intervention. These findings align with previous evidence summarized in a review [53], which reported that CBT-I and sleep medication exhibit comparable short-term effects. This suggests that CBT-I can achieve meaningful improvements in insomnia symptoms irrespective of medication use.

CBT-I is generally considered a first-line treatment due to its superior safety and sustained effectiveness compared with sleep medications, which often fail to maintain their effects after discontinuation [53]. These characteristics highlight the potential of CBT-I as a key strategy for long-term management of insomnia.

Moreover, a study suggests that combining CBT-I with sleep medication may provide benefits in terms of faster symptom relief during the initial stages of treatment [53]. However, such combined therapy may compromise the long-term benefits of CBT-I, underscoring the need for cautious management. This indicates that while combining medication with CBT-I might be beneficial for initial symptom relief, transitioning to CBT-I as a stand-alone treatment may be one of the effective approaches.

As this study primarily focused on short-term effects over an 8-week period, further research is needed to evaluate the long-term impacts of CBT-I. Future studies with extended follow-up periods could provide valuable insights into the durability of CBT-I’s effects and the potential risks of symptom recurrence. Such research would also help clarify the advantages and limitations of combining CBT-I with medication, offering evidence-based guidance for optimizing treatment strategies.

### Integrating ICT-Based CBT-I Into Community Health Systems

This study suggests that despite being provided in a non–face-to-face manner, the program can successfully alter participants’ perceptions of sleep. Although CBT-I is a highly effective treatment for insomnia, its limited availability and accessibility mean that only a small number of insomnia patients benefit from it [54]. Therefore, Smart Sleep using ICT may offer an opportunity to provide CBT-I to a larger number of insomnia patients, and it is thought that it can contribute to the improvement of insomnia in the community. A study highlighted the effectiveness of in-person therapist-led programs in CBT-I due to their structured nature and personalized support [48]. However, the results of this study suggest that similar outcomes can be achieved in a non–face-to-face environment when mobile apps are designed with interactive features and tailored support mechanisms. For example, the real-time consulting and individualized feedback provided through the Smart Sleep app in this study may have replicated some of the benefits associated with therapist-led interventions, thereby contributing to the high retention and adherence rates observed among participants. This demonstrates the potential of ICT-based approaches to broaden the accessibility of CBT-I while maintaining its effectiveness. Particularly for older adults, who may require flexible and user-friendly digital solutions, such approaches could serve as an effective alternative to traditional in-person therapies.

However, it is necessary to use existing infrastructure for scalability and integrate it into local public health systems to reduce logistical barriers. To achieve scalability, leveraging existing infrastructure is not only cost-effective but also essential for streamlining implementation processes. Integrating these efforts into local public health systems can further enhance operational efficiency and reduce logistical barriers, ensuring that solutions are accessible and sustainable within diverse community settings. This approach is particularly important as initial feedback revealed that concerns about potential costs led some individuals to decline participation in the study. By relying on existing infrastructure and public health resources, it becomes possible to reduce financial barriers and promote greater inclusivity in implementation. Future efforts should focus on evaluating these approaches to ensure the program’s broad applicability and sustainable impact.

### Limitations

This study was conducted in a specific group (ie, community-dwelling older adults), and the sample size was small, which limited the generalizability of the results to all older people. To ensure broader applicability, future research should involve diverse regions and larger sample sizes. In addition, while baseline medical conditions were assessed in this study, they were not explicitly included in the main analysis, which may have influenced the observed outcomes. Participants in this study may have had relatively higher technological literacy, as they voluntarily engaged in an ICT-based program. Moreover, participants were recruited from a local public health center, which may have introduced selection bias by including individuals already engaged in health services. This potential selection bias underscores the importance of including individuals with varying levels of tech literacy and service access in future studies.

Second, insomnia is a chronic condition with a risk of recurrence, making continuous management and monitoring necessary, even after short-term treatment. While CBT-I was effective for insomnia in this study, symptoms may recur because of factors such as stress or life changes. Therefore, long-term follow-up is essential to assess the sustained effects of the intervention.

Finally, the older might require more time to adapt to program usage than the younger age groups. In this study, an experiment was conducted while the researcher continued to educate participants on how to use the program. In the case of the older, programs that are easy to use, either by telephone coaching, as in this study, or by being very interactive, should be developed, and ongoing efforts should be made to improve and evaluate the usability of treatment programs.

### Conclusions

The results of this study showed that Smart Sleep is an ICT-based CBT-I that can provide effective non–face-to-face treatment for older adults with insomnia. Individuals who participated in the Smart Sleep program were able to effectively improve their sleep and most related measures through various treatment contents such as sleep assessment, sleep diary, and sleep monitoring via wearable devices, sleep restriction, stimulus control, cognitive therapy, sleep hygiene education, relaxation techniques, sleep knowledge education, exercise programs, and telephone coaching. The program is easy to use and has an intuitive menu structure, which makes it easy for the older to use, and the telephone coaching is thought to have contributed to the high usage rate. In the future, to effectively provide Smart Sleep to the older adults in the community, it is recommended to distribute it through community welfare or public health centers to improve the sleep health of many older people.

## Appendix

Multimedia Appendix 1 CONSORT 2010 checklist.

## Abbreviations

CBT-I: cognitive behavioral therapy for insomnia
DBAS: Dysfunctional Beliefs and Attitudes About Sleep
GDS: Geriatric Depression Scale
ICT: information and communication technology
ISI: Insomnia Severity Index
ISI-K: Korean version of the Insomnia Severity Index
K-DBAS-16: Korean version of the Dysfunctional Beliefs and Attitudes About Sleep
NNT: number needed to treat
PSQI: Pittsburgh sleep quality index
PSQI-K: Korean version of the Pittsburgh Sleep Quality Index
SGDS-K: Korean version of the short geriatric depression scale
QoL: quality of life
